# Perception of Vulnerability and Ruminant Thoughts about COVID-19 in Spanish Students †

**DOI:** 10.3390/ejihpe12100101

**Published:** 2022-10-01

**Authors:** José-María Figueredo, Cristina García-Ael, Gabriela Topa

**Affiliations:** 1 International School of Doctorate, National Distance Education University (UNED), 28040 Madrid, Spain; 2 Department of Social and Organizational Psychology, Faculty of Psychology National Distance Education University UNED, 28040 Madrid, Spain

**Keywords:** COVID-19, rumination, academic performance, perceived vulnerability to disease

## Abstract

The current situation in schools in relation to COVID-19 can generate a decrease in academic performance due to factors intrinsic to students. Therefore, rumination about COVID-19 could interfere with students’ attention, resulting in a decrease in their academic performance. Therefore, the objective of this study was to explore the relationship between the perception of vulnerability to the disease and rumination about COVID-19 from a cross-sectional sample of post-compulsory education students. The differences in the perception of vulnerability to disease and rumination in different groups were analyzed, separated by gender. Our data suggest a positive relationship between the perception of vulnerability to the disease and ruminants’ thoughts about COVID-19 (r = 0.29). Gender differences are significant, with women having higher scores than men in both variables.

## 1. Introduction

At the beginning of 2020, the disease caused by the coronavirus (COVID-19) broke into our lives. The continuous news about COVID-19, the global economic crisis, and the measures adopted by the governments of different countries to stop the pandemic have resulted in an increase in feelings of anxiety and depression in the general population [1]. These feelings of anxiety and depression cause an increase in intrusive or ruminant thoughts [2]. Ruminant thoughts are intrusive, repetitive, passive, and automatic thoughts about negative personal concerns and about the possible implications of a negative state [3]. Based on this literature, we propose the hypothesis (H1): the level of ruminant thoughts about COVID-19 will be medium or high among post-compulsory education students, (we set 1–2 as a low score, 3–4 as a medium score, and 5 as a high score on a five-point scale).

The tendency to have ruminant thoughts depends on individual differences in the cognitive processes that control information processing [4]. Thus, rumination will be associated with a decrease in the ability to inhibit information that is not relevant and distracting [5,6], and with other non-inhibitory forms of control [7], such as excessive autofocus [8]. However, the level of rumination may be associated with other individual traits [8].

The fear of being infected by COVID-19 is positively related to depression, anxiety, and the perception of vulnerability to the disease [9]. The perception of vulnerability to disease can be defined as personal beliefs about the susceptibility of contracting contagious diseases and the emotional distress associated with this possible contagion [10]. Perception of vulnerability to the disease is positively related to disgust [11] and the rejection of people who pose a threat of infection [12]. Based on these findings, and taking into account that the relationship between the perception of vulnerability to the disease and ruminant thinking about COVID-19 has not been examined, we propose our second hypothesis (H2): the perception of vulnerability to COVID-19 will be directly and positively associated with the level of rumination on COVID-19.

Researchhasshown that there are gender differences in the levels of ruminant thoughts, being higher in women than in men [13], and that these repetitive negative thoughts are related to depression and anxiety during adolescence [14]. In addition, an investigation [15] documented that more women use rumination as a strategy of emotional regulation in learning. Finally, a recent study [16] highlights the impact of the pandemic on the mental health of students in terms of stress, anxiety, and depression, finding a higher prevalence of negative symptoms in women. However, rumination about COVID-19 and the perception of vulnerability to the disease were not included in this study. On the other hand, other research has found that women perceive themselves as more vulnerable to contracting the disease in general [17], and to COVID-19 in particular [18]. Based on these findings, we propose the third hypothesis (H3): the level of rumination about COVID-19 and the perception of vulnerability to COVID-19 will be higher in women than in men.

Examining the personal factors that trigger rumination in academic contexts seems to us to be a particularly relevant topic for several reasons. Ruminant thoughts cause a variety of negative symptoms [19]. For example, ruminant thoughts are positively associated with stress [20], procrastination in students [21], and burnout at work [22], and negatively associated with motivation and concentration [23], and with vigor at work [22]. Ruminant thoughts continuously consume cognitive and energy resources, causing interference in attention [24], concentration, and motivation [23], and consequently, non-task-related rumination can cause a decrease in the individual’s performance in academic tasks [25].

## 2. Method

### 2.1. Participants

The sample consisted of 149 post-compulsory education students (n women = 129; 86.6%), all of them older than 18 years, with a mean age of 22.91 years (SD = 6.84). At the time of research, 111 subjects (75.4%) were studying vocational training, 33 (22.1%) were working on their university degree, 3 (2%) on their master’s degrees, and 2 were in doctoral studies (1.3%). A total of 75.4% of the participants were students at the Alhadra Professional Training Institute (Almería, Spain). The remaining were students at the University of Almería (Spain), while 24.2% of the subjects combined their studies with work, and 10.7% were looking for a job. Regarding the level of studies, the majority had a high school education(*n* = 82; 55%) or professional training (n = 48; 32.2%), while the rest were university graduates (*n* = 19; 12.8). A total of 13.4% of the subjects reported having a chronic or long-term illness.

### 2.2. Instruments

Participants rated their responses on a five-point scale (1 = strongly disagree, 5 = strongly agree).

Rumination about COVID-19. Rumination about COVID-19 was measured with a modified version of the rumination subscale of the Cognitive Regulation of Emotions questionnaire [26]. We followed the procedure usedby Bakker & van Wingerden [22]. In this way, each item on the scale made specific reference to COVID-19.For example, “This week, I was often thinking about how I feel about the coronavirus”. Cronbach’s alpha coefficient was 0.90. The possible scores on this subscale rangefrom 1 to 5, since mean scores are used for each participant. We established a low score of 1–2, a medium score of 3–4, and a high score of 5. Studies prior to the COVID-19 pandemic [26], carried out with a non-clinical sample from the general population and composed of 582 young people, show a mean of 7.89 (*SD* = 3.52) on a scale whose maximum score is 20. We obtained a higher mean (*M* = 12.65, *SD* = 4.45). We must bear in mind that we obtained the data in the midst of the COVID-19 pandemic.

Perception of vulnerability to disease. Perception of vulnerability to the diseasewas measured with the Perceived Vulnerability to Disease Scale (PVD) [27], validated in Spanish by Magallares et al. [10], in its Spanish short version [10]. PVD is made up of two factors: perceived infectivity and aversion to germs. The perceived infectivity subscale refers to personal perception of the risk of being infected, while the germ aversion subscale examines rejection or fear of germs. The reliability of the general scale was acceptable (∝ = 0.7). The possible scores on the PVD scale range from 1 to 5, since mean scores are used for each participant. We established a low score of 1–2, a medium score of 3–4, and a high score of 5. Studies prior to the COVID-19 pandemic carried out on Spanish university students [10] show a mean of 2.25 (*SD* = 1.30). Our results show a higher mean (*M* = 3.03; *SD* = 0.6), taking into account that the data was obtained in the midst of the COVID-19 pandemic.

### 2.3. Process

The sample was obtained by non-probability sampling. The participants were informed of the objectives of the study and the anonymity of the collected data. Participation was voluntary, and the participants in the final sample gave their consent to participate in the study and could withdraw from the study whenever they wanted. After expressing their consent to participate in the research, the participants individually filled in the questionnaire scales (Rumination, PVD). The format of the questionnaires was online. Data were collected during the month of January 2021.

### 2.4. Analysis of Data

Descriptive analyses and correlations were carried out. Subsequently, a linear regression analysis was carried out between the variable rumination on COVID-19 and the perception of vulnerability to the disease. The Student’s t-test for independent samples was performedto measure gender differences.

## 3. Results

### 3.1. Descriptive and Correlation Analysis

First, a bivariate correlation analysis was performed to find out the relationships between the variables. In all cases, the assumptions of independence, collinearity, and homoscedasticity were met (IVF was less than 5, and the tolerance index was greater than 0.2 in all cases, so our data verified the assumption of non-collinearity between the variables).

As can be seen in Table 1, rumination on COVID-19 was directly and positively associated both with the perception of vulnerability to the disease (*r* = 0.29) and with the perceived infectivity (*r* = 0.24) and aversion to germs (*r* = 0.20).

As can be seen in Table 2, 42.3% of the participants had a medium level of rumination, while 51.7% had a high level of rumination.

### 3.2. Linear Regression

In order to delve into the relationships between rumination on COVID-19 and the perception of vulnerability to the disease, we calculated a regression analysis between the variables. The results confirm that the level of rumination on COVID-19 can be predicted through the level of susceptibility to the disease through the equation (y = 1.78 + 0.45X). Confidence intervals were estimated using the bootstrapping technique with 5000 subsamples (β = 0.45, *p* < 0.05; 95% CI [0.20, 0.69]) (Table 3).

Subsequently, we also performed a linear regression analysis between the same variables, differentiating between genders. Results for women were similar to those for the full sample (y = 1.95 + 0.41x, *p* < 0.05) (Table 4) However, it was not possible to build a linear regression model for men, since *p* was greater than 0.05. This was probably because the sample of men was too small.

### 3.3. Student’s t-Test

To analyze gender differences in COVID-19 rumination and the perception of vulnerability to the disease, the Student t-test was performed for independent samples. The results confirmed that the level of rumination about COVID-19 (*p* = 0.04) and the level of perception of vulnerability to the disease (*p* = 0.02) was higher in women than in men.

Table 5 shows the means, standard deviations, and response ranges for the entire sample of participants, for men and for women. Women obtained significantly higher scores in rumination (*M* = 3.16, *SE* = 0.09) than men (*M* = 2.7, *SE* = 0.25) (*t* = −2.01, *p* < 0.05). Women also obtained significantly higher scores in the perception of vulnerability to COVID-19 (*M* = 3.03, *SE* = 0.06) than men (*M* = 2.06, *SE* = 0.10) (*t* = −2.34, *p* < 0.05).

## 4. Discussion

The general objective of our research was to explore the relationships between rumination about COVID-19 and the perception of vulnerability to the disease. Our results confirm that the degree of ruminant thinking about COVID-19 in the student population is medium–high. Likewise, it was confirmed that there is a direct and positive relationship between ruminant thoughts about COVID-19 and the perception of vulnerability to the disease. Our findings are consistent with some research examining certain negative psychological reactions that affect people’s well-being during times of infectious epidemic crises [16,18,28,29,30,31,32,33,34].

During the pandemic, ruminating thoughts about COVID-19 have been common among students [29,30,32,33,34]. In addition, rumination about COVID-19 is associated with the consequences of stress [29] and psychological distress [33], negatively influencing the mental well-being of university students [32]. In fact, ruminating thoughts may be one of the factors contributing to college students’ susceptibility to depressive symptoms during the COVID-19 pandemic [30,31,32,33,34], so much so, that the results of the meta-analysis carried out by Li et al. [31] showed that the prevalence of depression or anxiety increased significantly in university students during the COVID-19 pandemic. Similarly, in a longitudinal study carried out with university students by Kavvadas et al. [16], high levels of anxiety, stress, and depression were observed;these levels affected women more than men during the second year of the pandemic.

In all these investigations, rumination is analyzed as an antecedent factor of psychological distress, but the antecedents of ruminant thoughts are not investigated. In our research, we analyzed a personal variable that can have effects on rumination levels about COVID-19, such as the perceived vulnerability to infectious diseases.

Analyzing rumination about COVID-19 and its background is important for the student population because, in addition to causing psychological discomfort, it can cause concentration difficulties [29] and, consequently, poor academic performance [35]. Our findings advance a better understanding of the formation of rumination during the COVID-19 pandemic on the most vulnerable groups, such as the female student population.

Our results reflect gender differences. Rumination levels about COVID-19 were higher in women. These results are consistent with other research that analyzes the relationship between rumination and gender differences [13,14,15]. Similarly, women presented a greater perception of vulnerability to the disease than men. The results are consistent with other research that analyzes the role of gender in the perceived vulnerability to infectious diseases [17,18,27].

These results do not imply, in any case, that women have higher rates of COVID-19 infection in the place where this study was carried out; we did not have enough information to contrast this assumption. On the other hand, higher levels of rumination about COVID-19 and a greater perception of vulnerability to COVID-19 could act as a protective mechanism against COVID-19 infection in young women [18], such as the women in our sample. This is explained by the existence of a unique integrated compensatory biological/behavioral immune system [17]. In fact, women are more concerned about infectious signs when they are of childbearing age, which occurs in young women [36], like those in our sample.

### 4.1. Limitations

Our research has certain limitations. The first limitation would be related to the use of self-report measures. Self-report measures are not very objective and may be biased by the subject’s capacities for introspection, self-observation, self-assessment, memory, and the emotional and motivational state at the time of the response. Thus, for example, lack of motivation can cause a subject to respond quickly and not accurately. However, there is no other method to measure ruminant thoughts or the perception of vulnerability to disease.

The second limitation would be related to the sample. Our sample was made up of post-compulsory education students. In addition, our sample was small, which favors imprecision in the results and difficulties in finding significant results. It would be important to examine different groups of students, of different educational levels, and use larger samples. On the other hand, it is necessary to comment that there are a series of additional variables related to social status that can shape individuals’ perceptions of danger and vulnerability, including marital status, parenting status, residency (e.g., dorm, alone, with family), financial independence/dependence, and type of professional training. In our case, all the participants studied professional training related to social and community services, but we did not obtain information related to the other variables.

The final limitation is the cross-sectional nature of the research design. The hypothesized casual relationships should be confirmed in future longitudinal studies. Despite these limitations, our findings open a series of relevant avenues for future research.

### 4.2. Future Research

Future research should examine the impact of ruminant thoughts about COVID-19 on students’ academic performance. For this, the same procedure that was carried out in this studycould be followed to assess the effect of goal-directed rumination on academic performance [37]. Thus, it will be possible to complete self-report questionnaires on COVID-19 rumination (or other infectious diseases) at different times (one, two, and threeweeks before a test or exam) and relate the performance of the students with their level of rumination. A laboratory experiment could also be carried out in which the participants (experimental group) are asked to perform a complex task (for example, the Raven matrices) after having induced a certain level of rumination (for example, by saying that previously, that same place was occupied by another participant infected with COVID-19 or another serious infectious disease).

Likewise, it would be necessary to deepen the knowledge about gender differences in terms of ruminant thoughts and the perception of vulnerability.

Regarding academic performance, it would be necessary to measure the effect due to the interference caused by ruminant thoughts about COVID-19. For this, it would be necessary to study the simultaneous effect of other variables that have currently broken into teaching contexts at the time of pandemic, such as new methodologies that imply less face-to-face attendance and less direct supervision of students by teachers, in addition to the classic variables (study time, perceived self-efficacy, students’ engagement). Finally, this research could be replicated with measures specifically designed for COVID-19, such as the COVID-19 Perceived Scale (CPRS) [38] and the COVID-19 Rumination Scale (C-19RS) [39]. These scales were not available at the time that the data for our investigation were collected. However, we think that our findings could be extended to other future epidemic crises, different from COVID-19.

### 4.3. Practical Implications

The results of this research confirm that the level of rumination on COVID-19 is medium–high among students of post-compulsory education, especially women, which depends, in part, on the perception of vulnerability to the disease. The elevated levels of perceived vulnerability in young women can be explained by the existence of a unique integrated compensatory biological/behavioral immune system [17]. However, we cannot forget the possibility that men and women may have different types of concerns related to their health as well as their place in the social structure as mothers/fathers and as different types of workers.

Given that these circumstances could have a negative effect on attention, concentration, motivation, and, in short, on academic performance and student engagement [15], it would be advisable to introduce systems that counteract the effect of rumination on cognitive performance and student engagement. In this sense, various studies [30,33,40] have confirmed that mindfulness practices counteract the negative effects of rumination and have a positive effect on well-being and the capacity for learning in university students. In addition, interventions that help college students ensure adequate rest and physical exercise could have a positive effect on rumination [30]. On the other hand, employing a playful job design would be helpful in dealing with ruminant thoughts about COVID-19. This has been demonstrated in the workplace by Bakker & van Wingerden [22]. Similarly, playful learning can have positive effects on the mental health of students and teachers in the global context of a pandemic [41]. In an article by Whitton [42], the tools, techniques, and tactics of playful learning are described, mainly in adulthood, such as learning through the design of escape rooms. In this way, when implementing distance learning (which has increased during the pandemic in much of the world), it would be good to design interesting, fun, and challenging learning activities using applications that gamify teaching, such as the web application Classcraft (https://www.classcraft.com/) (accessed on 1 May 2021) or the Genially app (https://genial.ly/) ((accessed on 1 May 2021) with which you can create a wide variety of interactive games. Active use of these tactics for academic work can create a more interesting experience. The states generated by this behavior can be used to cushion the impact of rumination on COVID-19. In short, it seems necessary to redesign academic work to make it more playful.

## 5. Conclusions

Our findings are consistent with the current understanding of the role of the perception of vulnerability to illness and dysfunctional thoughts. Adopting organizational measures that offer security to students against the possibility of being infected by COVID-19, as well as appropriate teaching strategies and methods, could preventan excess of ruminant thoughts and, consequently, result in less interference in attention.

## Figures and Tables

**Table 1:** Descriptive statistics and correlations.

Variables	*M*	*SD*	1	2	3	4
1. Rumination	3.16	0.04	1			
2. Infectibility	2.30	0.04	0.24 **	1		
3. Aversion	3.62	0.19	0.20 *	0.19 *	1	
4. Perception of vulnerability	3.03	0.6	0. 29 **	0.74 **	0.79 **	1

* *p* < 0.05; ** *p* < 0.01.

**Table 2:** Frequency of ruminant thoughts.

Scores	Frequency	Percentages
1–2	9	6.0
3–4	63	42.3
5	77	51.7

**Table 3:** Linear regression (men and women).

Effect of Susceptibility to Disease on…	F(1,147)	*R* ^2^	β	*SE*	*p*
Rumination	14	0.087	0.45	0.12	<0.001
Constant			1.78	0.38	<0.001

**Table 4:** Linear regression (women).

Effect of Susceptibility to Disease on…	F(1,127)	*R* ^2^	β	*SE*	*p*
Rumination	11	0.080	0.41	0.12	<0.005
Constant			1.95	0.4	<0.001

**Table 5:** Means, standard deviation, and ranges of the study variables.

Variables	Mean (*SD*) Total Group	Mean (*SD*) Men	Mean (*SD*) Woman	Observed Ranges
1. Rumination	3.16 (0.04)	2.7 (1.14)	3.23 (1.09)	1–5
4. Perception of vulnerability	3.03 (0.6)	2.6 (0.45)	3.09 (0.74)	1–5

## Data Availability

Data available upon request due to restrictions, e.g., privacy or ethical. The data presented in this study are available upon request from the corresponding author.
